# Prediabetes is an independent risk factor for sarcopenia in older men, but not in older women: the Bunkyo Health Study

**DOI:** 10.1002/jcsm.13074

**Published:** 2022-09-02

**Authors:** Hideyoshi Kaga, Yoshifumi Tamura, Yuki Someya, Hitoshi Naito, Hiroki Tabata, Saori Kakehi, Nozomu Yamasaki, Motonori Sato, Satoshi Kadowaki, Ruriko Suzuki, Daisuke Sugimoto, Ryuzo Kawamori, Hirotaka Watada

**Affiliations:** ^1^ Department of Metabolism and Endocrinology, Graduate School of Medicine Juntendo University Tokyo Japan; ^2^ Sportology Center, Graduate School of Medicine Juntendo University Tokyo Japan; ^3^ Sports Medicine and Sportology, Graduate School of Medicine Juntendo University Tokyo Japan

**Keywords:** sarcopenia, muscle strength, prediabetes, type 2 diabetes

## Abstract

**Background:**

Sarcopenia is a major cause of disability in the elderly. Although type 2 diabetes is a risk factor for increased sarcopenia, the relationship between prediabetes and sarcopenia has not been elucidated. We aimed to examine the relationship between sarcopenia and prediabetes.

**Methods:**

The design of this study is a cross‐sectional study. We evaluated glucose metabolism using the 75‐g oral glucose tolerance test and glycated haemoglobin, appendicular skeletal muscle mass, and hand grip strength in 1629 older adults living in an urban area of Tokyo, Japan. We investigated the frequency of sarcopenia in participants with normal glucose tolerance (NGT), prediabetes and diabetes. A multivariable logistic regression model was used to analyse the association between glucose tolerance and the prevalence of sarcopenia.

**Results:**

The mean age of participants was 73.1 ± 5.4 years. In men, 44.3% had NGT, 26.6% had prediabetes, and 29.1% had diabetes. In women, the distribution was 56.1%, 28.8% and 15.2%. The prevalence of sarcopenia was 12.7% in men and 11.9% in women. Logistic regression revealed that prediabetes and diabetes are independent risk factors for sarcopenia in men (prediabetes, odds ratio [OR] = 2.081 [95% confidence interval {CI}: 1.031–4.199]; diabetes, OR = 2.614 [95% CI: 1.362–5.018]) and diabetes, but not prediabetes, is an independent risk factor for sarcopenia in women (prediabetes, OR = 1.036 [95% CI: 0.611–1.757]; diabetes, OR = 2.099 [95% CI: 1.146–3.844]). In both sexes, higher age (men, OR = 1.086 [95% CI: 1.028–1.146]; women, OR = 1.195 [95% CI: 1.142–1.251]), higher body fat percentage (men, OR = 1.346 [95% CI: 1.240–1.461]; women, OR = 1.218 [95% CI: 1.138–1.303]) and lower body mass index (men, OR = 0.371 [95% CI: 0.299–0.461]; women, OR = 0.498 [95% CI: 0.419–0.593]) were independent risk factors for sarcopenia.

**Conclusions:**

Although we confirmed that diabetes mellitus is associated with sarcopenia in both sexes, prediabetes is associated with sarcopenia in men, but not in women.

## Introduction

The number of elderly people has increased worldwide. The ageing rate in Japan is the highest in the world. A serious social problem in an aged society is the increase in the number of elderly people who need long‐term care. Sarcopenia, defined as a decrease in skeletal muscle mass, strength and function, is recognized to be a common condition in elderly people who need long‐term care. Patients with diabetes mellitus have lower muscle strength than people without diabetes mellitus,^1^ and type 2 diabetes is associated with accelerated loss of leg muscle strength in elderly individuals.^2^ In fact, type 2 diabetes has been identified as a risk factor for sarcopenia.^3^ Because body mass index (BMI) of patients with diabetes mellitus in Asian countries is much lower than in other countries^4^ and low BMI is a strong predictor of sarcopenia,^5^ sarcopenia easily develops in elderly Asians with diabetes.^6^

Similar to diabetes, the prevalence of prediabetes, defined as elevated fasting plasma glucose, postprandial hyperglycaemia or both, is increased with ageing.^7^ Prediabetes is often diagnosed by health check‐ups and outpatient clinic in Japan, because annual blood glucose or HbA1c check are obligated to Japanese people over 40 years old. Because prediabetes is established as a risk factor for cardiovascular disease,^8^, ^9^ intensive prevention for cardiovascular disease is now recommended not only for diabetes but also for prediabetes in clinical guideline.^8^ In terms of sarcopenia, previous studies have revealed that hand grip strength adjusted by BMI^10^ or body weight^11^ is associated with prediabetes in East Asians. Therefore, prediabetes may be a risk for sarcopenia, and healthcare providers may need to pay attention to the development of sarcopenia in prediabetes as well as diabetes. However, the association between prediabetes and sarcopenia has not been clarified yet.

In this context, the purpose of the present study was to clarify the association between prediabetes and prevalence of sarcopenia in community‐dwelling elderly Japanese individuals.

## Research design and methods

### Study design and participants

The study subjects were all participants in the Bunkyo Health Study. The Bunkyo Health Study is a prospective cohort study designed to clarify whether muscle function (muscle mass, muscle strength and insulin sensitivity) is associated with multiple diseases that necessitate long‐term care,^12^ and we performed cross‐sectional analysis using the baseline data of the study. Briefly, in this study, we recruited elderly subjects aged 65–84 years living in Bunkyo‐ku, an urban area in Tokyo, Japan. Exclusion criteria consisted of pacemaker or defibrillator placement and diabetes requiring insulin therapy. Subjects underwent measurements over 2 days. On the first day, we evaluated cognitive function using questionnaires. We also evaluated muscle strength and physical performance. On the second day, after an overnight fast, we evaluated brain lesions with magnetic resonance imaging (MRI), bone mineral density with dual‐energy X‐ray absorptiometry, arteriosclerosis with the cardio‐ankle vascular index, abdominal fat distribution with MRI, and glucose tolerance with a 75‐g oral glucose tolerance test (75‐g OGTT). The study protocol was approved by the ethics committee of Juntendo University in November 2015 (Nos. 2015078, 2016138, 2016131, 2017121 and 2019085). This study was carried out in accordance with the principles outlined in the Declaration of Helsinki. All participants gave written informed consent and were informed that they had the right to withdraw from the trial at any time.

### Definition of glucose tolerance

We administered the 75‐g OGTT to all participants and measured haemoglobin A1c on the same day. According to the diagnostic criteria by the Japan Diabetes Society, diabetes was defined as fasting plasma glucose ≥126 mg/dL, 2‐h glucose level after 75‐g OGTT ≥ 200 mg/dL, haemoglobin A1c ≥ 6.5% or use of oral hypoglycaemic agents.^13^ Normal glucose tolerance (NGT) was defined as fasting plasma glucose <110 mg/dL, 2‐h glucose level after the 75‐g OGTT < 140 mg/dL, and haemoglobin A1c < 6.5%. The remaining participants were defined as prediabetes.

### Muscle strength measurement

Grip strength was measured twice on each side using a hand grip dynamometer (T.K.K.5401, Takei Scientific Instruments Co., Ltd., Niigata, Japan). We used the average of the maximum values for each side for handgrip strength. We evaluated isokinetic muscle strength of knee extensors and flexors using a dynamometer (BIODEX system 3 or 4, Biodex Medical Systems, Upton, NY, USA). Isokinetic peak torque of knee extensors and flexors was measured at an angular velocity of 60°/s. During the test, participants were encouraged to exert maximal muscle force.

### Physical function tests

We administered other physical function tests, including a one‐leg standing test (balance test), which measured the duration that subjects could stand on one leg with their eyes open; maximum gait speed (10‐m walking test); and a Timed Up and Go test (TUG), which measures the time that it took for a participant to stand up from a seated position, walk 3 m, turn, walk back and sit down again.

### Other measurements

Appendicular skeletal muscle mass (ASM) was evaluated with bioelectrical impedance analysis (InBody770, InBody Japan Inc., Tokyo, Japan). Physical activity was evaluated using the International Physical Activity Questionnaire, which assesses different types of physical activity, such as walking and both moderate‐ and high‐intensity activities. Nutritional status was evaluated using a brief self‐administered diet history questionnaire that contained 58 items about fixed portions and food types. Hypertension was defined as systolic blood pressure ≥ 140 mmHg, diastolic blood pressure ≥ 90 mmHg or current use of antihypertensive medications. Dyslipidaemia was defined as low‐density lipoprotein cholesterol ≥ 140 mg/dL, high‐density lipoprotein cholesterol < 40 mg/dL, triglycerides ≥ 150 mg/dL, or current use of lipid‐lowering agents.

### Definition of sarcopenia

Sarcopenia was defined as weak hand grip (<28 kg for men and <18 kg for women) and low ASM (<7.0 kg/m^2^ for men and <5.7 kg/m^2^ for women) based on the definition of the Asian Working Group for Sarcopenia (AWGS) 2019.^14^

### Statistical analysis

Participants were divided into three groups (NGT, prediabetes and diabetes) for each sex. We used IBM SPSS Statistics for Windows, version 25.0. (IBM Corp., Armonk, NY, USA) for statistical analysis. Data are presented as means ± *SD* or number (%). There were considerable differences in body composition, muscle strength, physical function and glucose tolerance by sex. Therefore, all analyses were stratified by sex. Differences in means and proportions were tested using ANOVA and the *χ* ^2^ test. ASM, muscle strength and physical function differences were tested using ANCOVA with adjustment for age, BMI, % body fat, daily physical activity level, energy intake and cerebrovascular disease. Logistic regression was used to estimate odds ratios (ORs) and 95% confidence intervals (CIs) for the association between glucose tolerance and the prevalence of sarcopenia, with adjustments for potential confounders. In this study, three models were generated in regression analysis. Model 1 was adjusted for age. Model 2 was adjusted for variables in Model 1 plus BMI, % body fat, physical activity level and energy intake. Model 3 was adjusted for variables in Model 2 plus cerebrovascular disease. We determined the potential confounders based on prior knowledge, which is the most used method in epidemiology. Previous reports suggested that BMI, % body fat, daily physical activity level, energy intake and cerebrovascular disease are associate with muscle mass or muscle strength,^15^, ^16^, ^17^, ^18^ and several intervention studies have suggested causal relationships between the several factors raised and muscle mass or strength or physical function.^19^ All statistical tests were two‐sided with a 5% significant level.

## Results

### Characteristics by glucose tolerance status

The characteristics of the study participants are shown in Table 1. The mean age of subjects was 73.1 ± 5.4 years. Of 1629 participants, 156 participants (9.6%) were diagnosed with diabetes because they were taking oral hypoglycaemic agents. In the remaining 1473 participants, 2 participants had missing data on 2‐h glucose level after the 75‐g OGTT. One was diagnosed with diabetes, and the other was diagnosed with NGT based on fasting plasma glucose and haemoglobin A1c levels. In the Bunkyo Health Study, 187 (11.5%) were newly diagnosed with diabetes mellitus. In men, 44.3% of participants had NGT, 26.6% had prediabetes, and 29.1% had diabetes mellitus. In women, the distribution was 56.1%, 28.8% and 15.2%. Participants with prediabetes and diabetes were significantly older than those with NGT in each sex. Men in the Diabetes group had significantly lower energy intake than men in the prediabetes group. Women in the diabetes group had significantly shorter education duration than women in the NGT and prediabetes groups. Women in the diabetes group had significantly lower physical activity level than women in the NGT group. The NGT group had significantly lower BMI and % body fat than the prediabetes and diabetes groups in both sexes. The prediabetes group had higher subcutaneous fat area than the NGT group in both sexes. Visceral fat area increased as glucose tolerance worsened in both sexes.

**Table 1:** Clinical characteristics of the study participants by glucose tolerance status and sex

	Men				Women			
	NGT	Prediabetes	Diabetes	*P*	NGT	Prediabetes	Diabetes	*P*
N	304	183	200		528	271	143	
Age (years)	72.1 ± 5.4	73.6 ± 5.0*	73.8 ± 5.3*	<0.001	72.4 ± 5.5	73.9 ± 5.1*	74.8 ± 5.5*	<0.001
Education (years)	15.0 ± 2.3	14.9 ± 2.6	14.8 ± 2.6	0.476	13.3 ± 2.2	13.2 ± 2.2	12.6 ± 2.2*, †	0.001
Hypertension (%)	65.1%	76.5%	82.5%	<0.001	51.3%	70.8%	77.6%	<0.001
Dyslipidaemia (%)	51.0%	59.0%	67.5%	<0.001	59.1%	71.6%	83.9%	<0.001
IHD (%)	6.3%	2.2%	5.5%	0.123	2.5%	6.3%	2.8%	0.020
CVD (%)	4.9%	4.9%	13.0%	0.001	1.5%	2.6%	7.0%	0.001
PAD (%)	0.0%	0.0%	2.0%	0.007	0.0%	0.4%	0.7%	0.219
BMI (kg/m^2^)	23.0 ± 2.5	23.8 ± 2.8*	24.1 ± 2.9*	<0.001	21.6 ± 2.8	22.9 ± 3.3*	23.6 ± 3.6*	<0.001
% body fat (%)	23.0 ± 5.4	25.5 ± 6.1*	26.5 ± 5.7*	<0.001	29.5 ± 6.5	32.2 ± 6.8*	34.0 ± 7.4*, †	<0.001
SFA (cm^2^)	118.4 ± 42.0	134.8 ± 50.9*	133.5 ± 45.2	<0.001	156.4 ± 58.2	173.2 ± 62.9*	178.0 ± 67.7	<0.001
VFA (cm^2^)	79.2 ± 35.7	93.2 ± 38.2*	104.4 ± 42.0*, †	<0.001	60.5 ± 30.5	77.3 ± 36.2*	91.9 ± 39.1*, †	<0.001
Physical activity level (METs/hour/week)	49.2 ± 48.8	53.3 ± 76.4	40.7 ± 41.4	0.047	44.7 ± 45.9	38.2 ± 37.2	35.4 ± 34.2*	0.015
Dietary intake (kcal/day)	2120.9 ± 610.3	2216.1 ± 669.2	2059.2 ± 583.5†	0.045	1868.9 ± 555.0	1800.2 ± 522.8	1804.7 ± 559.1	0.174

*Note:* Data are means ± *SD*. *P* values for ANOVA or *χ* ^2^ test.

Abbreviations: CVD, cerebrovascular disease; IHD, ischaemic heart disease; METs, metabolic equivalents; NGT, normal glucose tolerance; PAD, peripheral artery disease; SFA, subcutaneous fat area; VFA, visceral fat area.

* <0.05 vs. NGT with Bonferroni correction.

† <0.05 vs. prediabetes with Bonferroni correction.

Table 2 shows blood test data in each glucose tolerance status group. Fasting plasma glucose, haemoglobin A1c and alanine aminotransferase levels increased as glucose tolerance worsened. Gamma glutamyl transpeptidase levels were significantly higher in the diabetes group compared with the NGT group in both sexes. Aspartate aminotransferase levels were significantly higher in the diabetes group compared with the NGT group in men. On the other hand, albumin, creatinine and 25‐hydroxyvitamin D levels, which are thought to positively affect muscle mass, did not differ among the groups.

**Table 2:** Blood test data of the study participants by glucose tolerance status and sex

	Men				Women			
	NGT	Prediabetes	Diabetes	*P*	NGT	Prediabetes	Diabetes	*P*
N	304	183	200		528	271	143	
Fasting plasma glucose (mg/dL)	94.9 ± 6.4	100.9 ± 9.3*	122.4 ± 23.0*, †	<0.001	92.2 ± 6.5	97.0 ± 8.7*	118.6 ± 24.2*, †	<0.001
haemoglobin A1c (%)	5.5 ± 0.3	5.7 ± 0.3*	6.5 ± 0.7*, †	<0.001	5.6 ± 0.3	5.8 ± 0.3*	6.6 ± 0.8*, †	<0.001
AST (IU/L)	22.4 ± 5.7	23.8 ± 9.1	25.2 ± 13.2*	.008	22.8 ± 5.7	23.6 ± 11.1	25.2 ± 15.8	0.121
ALT (IU/L)	17.8 ± 6.8	20.7 ± 9.6*	24.0 ± 18.8*, †	<0.001	17.2 ± 7.1	19.2 ± 12.3*	22.0 ± 13.3*, †	<0.001
γ‐GTP (IU/L)	35.3 ± 38.6	41.4 ± 43.2	46.9 ± 66.3*	.049	22.3 ± 18.0	25.0 ± 16.8	27.4 ± 18.2*	0.004
Creatinine (mg/dL)	0.96 ± 0.45	0.97 ± 0.54	0.94 ± 0.40	.081	0.67 ± 0.13	0.67 ± 0.13	0.70 ± 0.52	0.683
Alb (g/dL)	4.3 ± 0.4	4.3 ± 0.4	4.3 ± 0.4	.829	4.3 ± 0.4	4.3 ± 0.4	4.3 ± 0.3	0.959
25‐OHD (ng/mL)	20.3 ± 5.0	19.6 ± 5.1	19.8 ± 5.3	.299	18.4 ± 5.0	18.8 ± 5.1	17.9 ± 5.0	0.201

*Note*: Data are means ± *SD*. *P* value for ANOVA.

Abbreviations: Alb, albumin; ALT, alanine aminotransferase; AST, aspartate aminotransferase; γ‐GTP, gamma‐glutamyl transpeptidase; NGT, normal glucose tolerance; 25‐OHD, 25‐hydroxyvitamin D.

* <0.05 vs. NGT with Bonferroni correction.

† <0.05 vs. prediabetes with Bonferroni correction.

Table 3 shows the prevalence of sarcopenia and mean values of ASM, muscle strength and physical function parameters in each glucose tolerance status group. In this cohort, the prevalence of sarcopenia was 12.7% in men and 11.9% in women. ASM was comparable across groups in both men and women. After adjustment, in men, hand grip strength was lower in the diabetes group than in the NGT group, while hand grip strength was lower in the diabetes group than in the NGT and prediabetes groups in women. On the other hand, after adjustment, muscle strength in knee extensors and flexors, balance, maximum gait speed, and TUG scores were comparable across glucose tolerance status groups in both sexes.

**Table 3:** Comparison of arm and leg strength and physical function by glucose tolerance status and sex

	Men				Women			
	NGT	Prediabetes	Diabetes	*P*	NGT	Prediabetes	Diabetes	*P*
Sarcopenia (%)	8.6%	13.7%	18.0%	0.007^a^	10.6%	11.1%	18.2%	0.041^a^
ASM (kg/m^2^)	7.35 ± 0.62	7.33 ± 0.65	7.33 ± 0.68	0.967^c^	5.75 ± 0.55	5.86 ± 0.61	5.87 ± 0.53	0.089^c^
Low ASM (%)	31.6%	28.4%	31.5%	0.734^a^	45.5%	39.5%	42.0%	0.258^a^
Hand grip strength (kg)	33.1 ± 5.4	32.1 ± 6.1	31.1 ± 5.6*	0.048^b^	21.4 ± 3.4	21.4 ± 3.6	20.2 ± 3.7*, †	0.008^b^
Low hand grip strength (%)	17.1%	21.9%	30.5%	0.002^a^	14.2%	14.8%	29.4%	<0.001^a^
Muscle strength in knee extensors (Nm/kg)	1.53 ± 0.39	1.49 ± 0.39	1.41 ± 0.38	0.313^c^	1.28 ± 0.32	1.18 ± 0.31	1.09 ± 0.30	0.075^c^
Muscle strength in knee flexors (Nm/kg)	0.75 ± 0.25	0.73 ± 0.25	0.68 ± 0.23	0.368^c^	0.60 ± 0.19	0.55 ± 0.19	0.52 ± 0.19	0.540^c^
Balance (s)	57.2 ± 44.0	48.1 ± 44.0	38.5 ± 40.2	0.095^b^	58.3 ± 44.7	48.7 ± 41.2	38.0 ± 38.2	0.220^b^
Maximum gait speed (m/s)	2.01 ± 0.37	1.93 ± 0.39	1.90 ± 0.38	0.589^b^	1.87 ± 0.32	1.81 ± 0.35	1.74 ± 0.29	0.389^b^
Timed Up and Go test score (s)	6.3 ± 1.2	6.6 ± 1.6	6.8 ± 2.1	0.506^b^	6.6 ± 1.5	6.8 ± 1.5	7.2 ± 1.5	0.250^b^

*Note*: Data are means ± *SD*.

ASM, appendicular skeletal muscle mass; BMI, body mass index; NGT, normal glucose tolerance; Nm, Newton meters.

^a^ *P* value for *χ* ^2^ test.

^b^ *P* value for ANCOVA with adjustment for age, BMI, % body fat, daily physical activity level, energy intake and cerebrovascular disease.

^c^ *P* value for ANCOVA with adjustment for age, % body fat, daily physical activity level, energy intake and cerebrovascular disease.

* <0.05 vs. NGT with Bonferroni correction.

† <0.05 vs. prediabetes with Bonferroni correction.

Tables 4 and 5 show the ORs of each glucose tolerance group for sarcopenia in men and women, respectively. In men, after full adjustment, prediabetes and diabetes were each independently associated with a higher OR for sarcopenia when compared with NGT (prediabetes, 2.081 [95% CI: 1.031–4.199]; diabetes, 2.614 [95% CI: 1.362–5.018]). On the other hand, only diabetes was independently associated with increased ORs for sarcopenia in women (prediabetes, 1.036 [95% CI: 0.611–1.757]; diabetes, 2.099 [95% CI: 1.146–3.844]). In addition, age and % body fat were positively correlated, and BMI was negatively correlated with the prevalence of sarcopenia in both sexes.

**Table 4:** Associations between sarcopenia and glucose tolerance in men

Odds ratio (95% CI)			
	Model 1	Model 2	Model 3
Glucose tolerance			
NGT	1.000	1.00	1.00
Prediabetes	1.491 (0.823–2.698)	1.890 (0.946–3.775)	2.081 (1.031–4.199)
Diabetes	2.028 (1.168–3.523)	2.602 (1.326–4.971)	2.614 (1.362–5.018)
Age (per 1 year)	1.112 (1.064–1.163)	1.087 (1.030–1.147)	1.086 (1.028–1.146)
BMI (per 1 kg/m^2^)		0.375 (0.302–0.466)	0.371 (0.299–0.461)
% Body fat (per 1.0%)		1.346 (1.241–1.461)	1.346 (1.240–1.461)
Daily physical activity (per 1 MET/hour/week)		0.993 (0.985–1.000)	0.993 (0.985–1.000)
Energy intake (per 100 kcal)		0.991 (0.945–1.040)	0.990 (0.943–1.040)
CVD			3.470 (1.104–10.906)

*Note*: Model 1 was adjusted for age. Model 2 was adjusted for variables in Model 1 plus body mass index, % body fat, physical activity and energy intake. Model 3 was adjusted for variables in Model 2 plus CVD.

Abbreviations: BMI, body mass index; MET, metabolic equivalent; CVD, cerebrovascular disease.

**Table 5:** Associations between sarcopenia and glucose tolerance for women

Odds ratio (95% CI)			
	Model 1	Model 2	Model 3
Glucose tolerance			
NGT	1.000	1.00	1.00
Prediabetes	0.886 (0.546–1.440)	1.052 (0.623–1.775)	1.036 (0.611–1.757)
Diabetes	1.403 (0.825–2.287)	2.091 (1.142–3.829)	2.099 (1.146–3.844)
Age (per 1 year)	1.159 (1.114–1.207)	1.196 (1.142–1.252)	1.195 (1.142–1.251)
BMI (per 1 kg/m^2^)		0.500 (0.421–0.594)	0.498 (0.419–0.593)
% Body fat (per 1.0%)		1.216 (1.137–1.301)	1.218 (1.138–1.303)
Daily physical activity (per 1 MET/h/week)		0.998 (0.992–1.004)	0.998 (0.992–1.004)
Energy intake (per 100 kcal)		0.979 (0.937–1.022)	0.979 (0.938–1.022)
CVD			1.346 (0.400–4.529)

*Note*: Model 1 was adjusted for age. Model 2 was adjusted for variables in Model 1 plus body mass index, % body fat, physical activity and energy intake. Model 3 was adjusted for variables in Model 2 plus CVD.

Abbreviations: BMI, body mass index; MET, metabolic equivalent; CVD, cerebrovascular disease.

## Discussion

Sarcopenia and reduced muscle strength are associated with diabetes; however, these associations are not fully understood in prediabetes. The present study revealed that prediabetes is an independent risk factor for sarcopenia in men, but not in women.

The present study was the first to show that prediabetes is associated with sarcopenia in men. A previous study of a community‐dwelling cohort in the United Kingdom involving participants with NGT and prediabetes also showed an association between an increase in 2‐h glucose levels during 75‐g OGTT and a decrease in hand grip strength.^20^ In addition, ORs for poor physical function in men with diabetes mellitus or impaired glucose tolerance were 2.73 and 1.62 compared with NGT, respectively.^20^ Interestingly, these relationships were weaker and statistically non‐significant in women. The English Longitudinal Study of Ageing, which included 3404 older adults, also showed that diabetes predicted sarcopenia in men (OR: 2.43 [95% CI: 1.5–3.95]), but not in women (OR: 1.49 [95% CI: 0.83–2.68]).^21^ These data suggested that reduced glucose tolerance is associated with reduced muscle strength and poor physical function in men with and without diabetes mellitus. In terms of muscle mass, lower lean body mass with ageing was associated with incident diabetes in men, but not in women.^22^ These sex differences might be associated with different rates in muscle decline with ageing in males and females; declines in muscle mass^23^ and strength^24^ with ageing are greater in men than in women. However, in the present study, muscle strengths in lower limb were not reduced in both sexes. Our preliminary analysis revealed that the handgrip strength and muscle strength in knee extensor was only moderately correlated (men, *r* = 0.408, *P* < 0.001; women, *r* = 0.374, *P* < 0.001), suggesting these muscle strengths are differently regulated. In fact, the lower limb muscle is a weight‐bearing muscle and the hand grip muscle is a non‐weight‐bearing muscle; thus, these differences may modify the effects of prediabetes on muscle volume and strength.

We defined sarcopenia as the combination of both weak hand grip strength and low ASM in the present study.^14^ ASM and the prevalence of low ASM were very similar among the groups, whereas hand grip strength was significantly lower in subjects with diabetes than NGT. These data suggested that old adults with diabetes have low hand grip strength, even if they have something similar muscle mass. A previous study suggested that muscle quality, defined as muscle strength divided by corresponding muscle mass, is lower in patients with diabetes compared with individuals without diabetes.^1^ On the other hand, in men, post hoc analysis did not show significant difference in hand grip strength between NGT and prediabetes, and the prevalence of low hand grip strength was also comparable between NGT and prediabetes. However, prediabetes was independently associated with a higher OR for sarcopenia when compared with NGT in men. These results suggest that low ASM and low hand grip strength are more likely to be combined in prediabetes, which may account for the increased risk of sarcopenia in prediabetes. However, the mechanisms leading to sarcopenia in prediabetes and diabetes have not been elucidated yet. Hyperglycaemia reduces contractile function and force generation of muscle in mice.^25^ Insulin resistance is also associated with reduced muscle strength.^26^, ^27^ Diabetic neuropathy occurs in patients with diabetes or prediabetes^28^ and is associated with impaired muscle strength in patients with diabetes.^29^, ^30^ These factors might contribute to lower hand grip strength and higher prevalence of sarcopenia in prediabetes and diabetes. On the other hand, it could be also possible that sarcopenia cause prediabetes, because skeletal muscle is one of the major organs of insulin‐stimulated glucose uptake during postprandial state, and in fact, several studies showed that decreased muscle mass is associated elevated glucose level during OGTT.^31^, ^32^ Thus, the causal relationship between prediabetes and sarcopenia may be bidirectional; however, in the present study, the deterioration of glucose metabolism was not accompanied by a decrease in muscle mass.

Non‐Asian patients with diabetes have much higher BMI than Asian patients with diabetes.^4^ ASM is positively associated with BMI. Thus, the prevalence of sarcopenia could be lower in non‐Asian patients with diabetes compared with Asian patients with diabetes. In the present study, the prevalence of sarcopenia, defined as low hand grip strength and low ASM (AWGS 2019) was 12.7% and 11.9% in men and women, respectively. If we applied the cutoff values for grip strength and ASM from the European Working Group on Sarcopenia in Older People 2 (EWGSOP2),^33^ the prevalence of sarcopenia would be 10.5% and 6.8% in men and women, respectively (data not shown). Similarly, in a community‐dwelling Korean cohort, the prevalence of sarcopenia defined by hand grip strength and ASM was 14.4% in men and 6.4% in women with AWGS2019 cutoff values and 11.9% in men and 6.7% in women with EWGSOP2 cutoff values.^34^ In contrast, using the same hand grip strength and ASM cutoff values in the EWGSOP2 criteria, the prevalence of sarcopenia in a community‐dwelling elderly European cohort was 0.6–3.1% in men and 0–2.4% in women.^35^ Thus, the prevalence of sarcopenia in Asians could be much higher than in Europeans. It remains unclear whether prediabetes is associated with sarcopenia in non‐Asians.

Our data suggest a few clinical implications. As the present study suggested that sarcopenia develops even before diabetes develops, healthcare providers in hospital and community should recognize that prediabetes is a risk factor for sarcopenia and screen high‐risk old prediabetes for sarcopenia. In addition, healthcare providers should encourage high‐risk old prediabetes to prevent the development of sarcopenia by exercise and consumption of adequate amounts of protein. Of course, for efficient prevention of sarcopenia, the establishment of efficient intervention is required in the near future.

There are several limitations in this study. First, we did not evaluate peripheral neuropathy and duration of diabetes in subjects with previously diagnosed diabetes. A previous study showed that muscle quality is associated with duration of diabetes,^1^ and diabetes starting in midlife and longer duration of diabetes are significant risk factors for incident sarcopenia.^3^ Second, we defined sarcopenia based on hand grip strength and ASM. Thus, it remains unclear whether definite sarcopenia including physical performance is also associated with prediabetes. Third, the association between prediabetes and sarcopenia became significant with Model 3, while not significant in Model 1 and Model 2, suggesting that prediabetes may be a less clear and weaker risk factor for sarcopenia than diabetes. However, there were several confounding factors that weakened the association between prediabetes and sarcopenia without adjustment (downward bias). Finally, the present study is cross‐sectional and thus cannot establish causality.

In conclusion, the present study revealed that prediabetes is an independent risk factor for sarcopenia in men, but not in women, while diabetes is an independent risk factor for reduced hand grip strength and sarcopenia in both sexes. These associations were still significant after adjustment for many confounders, suggesting that elevated glucose level might be an important contributor to decreased muscle strength. Thus, our study suggested that prediabetic men and diabetic men and women are a high‐risk population of sarcopenia. Further studies are required to determine whether a combination of exercise and nutrition therapy can prevent sarcopenia in these high‐risk population.

## Funding information

This study was supported by Strategic Research Foundation at Private Universities (S1411006) and KAKENHI (18H03184) grants from the Ministry of Education, Culture, Sports, Science and Technology of Japan; Mizuno Sports Promotion Foundation; and the Mitsui Life Social Welfare Foundation.

## Conflict of Interest

The authors have nothing to disclose.
